# Ultra-Sensitive Humidity Sensor Based on Optical Properties of Graphene Oxide and Nano-Anatase TiO_2_

**DOI:** 10.1371/journal.pone.0153949

**Published:** 2016-04-21

**Authors:** Mahdiar Ghadiry, Mehrdad Gholami, C. K. Lai, Harith Ahmad, W. Y. Chong

**Affiliations:** 1 Photonics Research Center, University of Malaya, Kuala Lumpur, 50603, Malaysia; 2 Department of Chemistry, Marvdasht Branch, Islamic Azad University, P.O. Box 465, Marvdasht, Iran; VIT University, INDIA

## Abstract

Generally, in a waveguide-based humidity sensors, increasing the relative humidity (RH) causes the cladding refractive index (RI) to increase due to cladding water absorption. However, if graphene oxide (GO) is used, a reverse phenomenon is seen due to a gap increase in graphene layers. In this paper, this interesting property is applied in order to fabricate differential humidity sensor using the difference between RI of reduced GO (rGO) and nano-anatase TiO_2_ in a chip. First, a new approach is proposed to prepare high quality nano-anatase TiO_2_ in solution form making the fabrication process simple and straightforward. Then, the resulted solutions (TiO_2_ and GO) are effortlessly drop casted and reduced on SU8 two channels waveguide and extensively examined against several humid conditions. Investigating the sensitivity and performance (response time) of the device, reveals a great linearity in a wide range of RH (35% to 98%) and a variation of more than 30 dB in transmitted optical power with a response time of only ~0.7 sec. The effect of coating concentration and UV treatment are studied on the performance and repeatability of the sensor and the attributed mechanisms explained. In addition, we report that using the current approach, devices with high sensitivity and very low response time of only 0.3 sec can be fabricated. Also, the proposed device was comprehensively compared with other state of the art proposed sensors in the literature and the results were promising. Since high sensitivity ~0.47dB/%RH and high dynamic performances were demonstrated, this sensor is a proper choice for biomedical applications.

## Introduction

Monitoring humidity is essential is many industries and applications [1]. It is vital in medical and chemical industries to monitor patients breathing profile and control the chemical processes [2, 3]. In addition, it is essential in aerospace and civil engineering. As an example, it is checked regularly in huge structures like bridges and planes to assess the risk of leakage due to corrosion [4]. Besides, it is influential parameter in health of workers and product quality in food engineering [5]. Consequently, it has been a hot research topic for last decades and different types of humidity sensors including optical [6–8], resistive [9], thermal conductivity and capacitive [10] sensors have been designed and reported in the literature [11].

Depending on the applications, a humidity sensor requires high sensitivity, high speed (low response time) and noise immunity [10, 12]. One way to simply meet these requirements is to utilize optical devices. Geometry simplicity and small dimension of optical sensors bring about implementation of light-weight devices in a way that it is possible to simply insert them in medical probes and construction material [2]. Additionally, the sensitivity and detection range of optical devices can be significantly higher than that of the other conventional devices such as electrochemical sensors. These promising characteristics, make the optical devices pertinent alternatives for applications where high speed, low noise and high accuracy are appealed [13].

Furthermore, in situations, where humidity changes rapidly, sensors with low response and recovery time are needed. Such applications includes process control in industry, meteorology and variety of other medical applications such as human breath monitoring [14, 15]. This shows the need for fast, highly sensitive and low cost humidity sensors.

Therefore, in this research, an optical method is proposed to implement a humidity sensor using nano-anatase TiO_2_ particles and reduced graphene oxide (rGO). These two material have been employed in a variety of sensing applications such as gas and humidity sensing and promising results have been reported [16–19]. The basic idea is to measure the relative humidity using the difference between refractive indices (power loss) of rGO and TiO_2_. In material preparation, a new approach is engaged to prepare TiO_2_ nanoparticles with very small particle size around 5 nm in solution form making the fabrication low-cost, effective and smooth.

## Material Preparation

### Graphene Oxide

**Fig 1:**
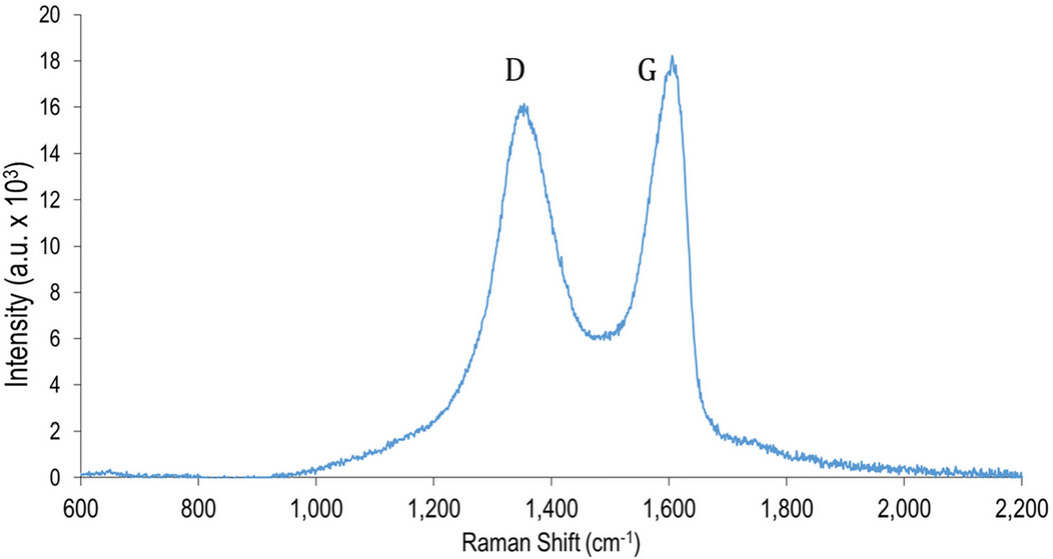
The Raman spectra of GO indicating D and G peaks.

GO solution was prepared by an improved version of Hummer’s method according to [20]. The GO solution in de-ionized water as the solvent has a concentration of about 1 μg/μL. Raman spectra of the produced GO displayed in Fig 1, clearly shows D and G peaks indicating the quality of the material according to argument reported in [21].

### Nano-Anatase TiO_2_ powder

A new approach is presented here to prepare TiO_2_ NPs. Initially 2 ml of aetylacetone solution was mixed with 2 ml titanium isopropoxide in a 10 ml measuring cylinder, then the prepared solution was mixed with 40 ml of absolute ethanol in a beaker under vigorous stirring at room temperature. Later, it was added to a solution containing 10 mL of 5% (W/V) of urea dropwise in a beaker under stirring at room temperature. The final solution was pale yellow in color with a pH value of 5.6. After vigorous stirring of this solution for one hour, the whole mixture was transferred into a 120 ml Teflon-lined stainless steel autoclave for 18 h at 150°C and cooled to room temperature naturally. The produced solution was centrifuged with DI water and absolute ethanol for several times. This residue was dried for 3 h at 80°C. The resulted powder was grinded to obtain fine powder which was used for preparation of nano-anatase TiO_2_ solutions of different concentrations. In this experiment, solutions of 0.01% (Sample 1), 0.02% (Sample 2), 0.04% (Sample 3) and 0.08% (Sample 4) (W/V) of nano-anatase TiO_2_ were prepared from the solid powder.

### Field emission scanning electron microscopy (FESEM)

**Fig 2:**
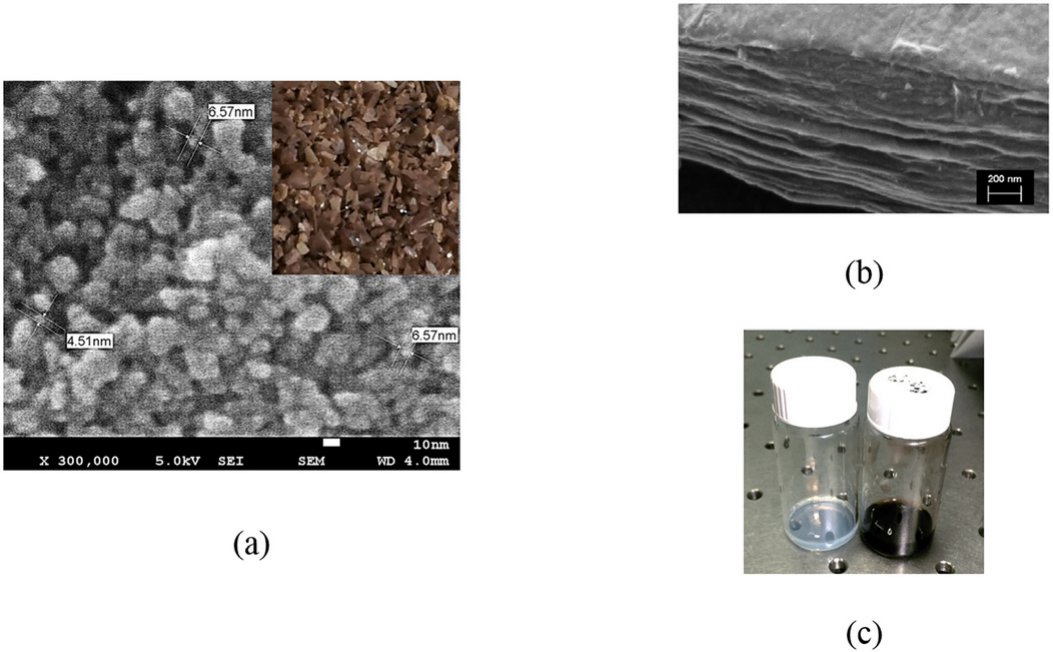
The FESEM images of titanium dioxide nano particles (a); same material in form of powder (subset); layered structure of GO (b); prepared TiO_2_ and GO solution to be drop casted on the waveguide (c).

Field emission scanning electron microscopy (FESEM) was used in order to perform structural and morphological analysis of the prepared TiO_2_ powder and rGO. Fig 2(a) and 2(b) illustrate the FESEM image of TiO_2_ nanoparticles and TiO_2_ powder (subset) and layered structure of the synthesized GO respectively. In addition, in Fig 2(c), TiO_2_ (silver-color solution) and rGO (dark brown) are seen in solution form.

**Fig 3:**
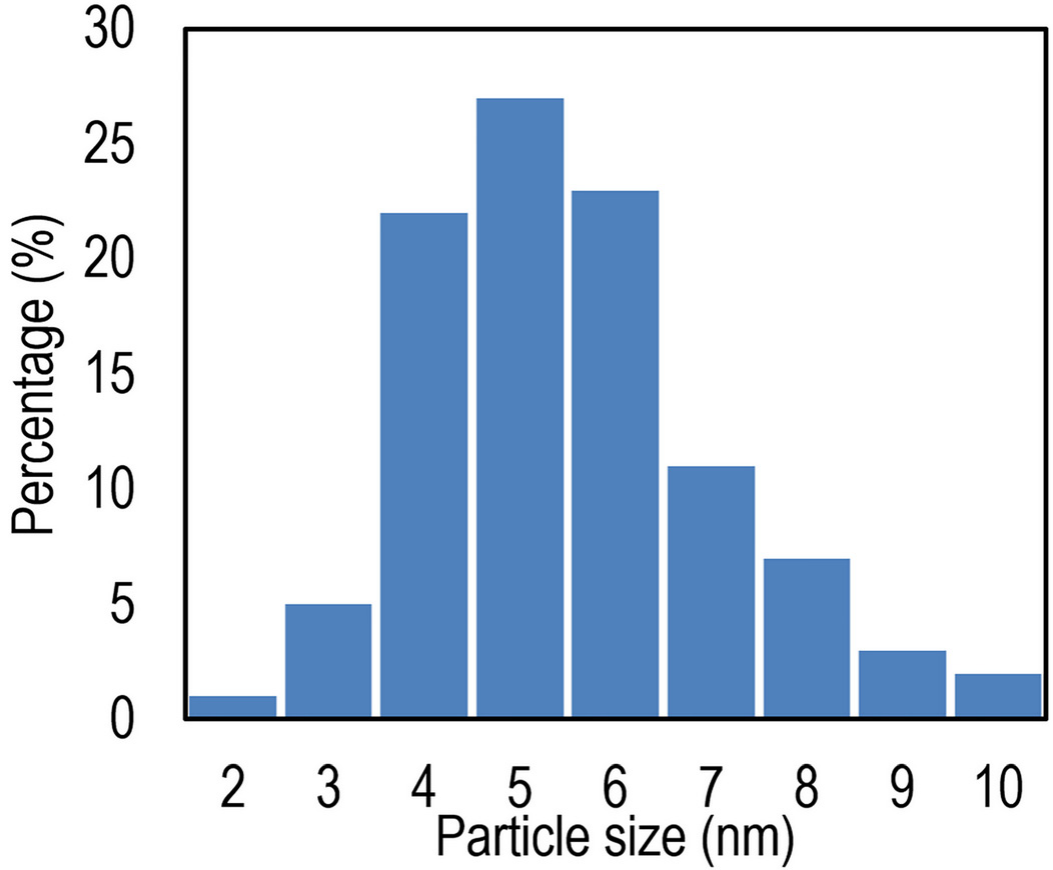
Normalized size distribution of the prepared TiO_2_ nano particles.

Result of size distribution analysis of the TiO_2_ sample is depicted in Fig 3. Accordingly, it can be recognized that most of particle size have a diameter less than 10 nm and maximum concentration is seen on 5 nm while in commercial TiO_2_, the average particle size is around 20 nm.

### X-ray diffraction (XRD)

**Fig 4:**
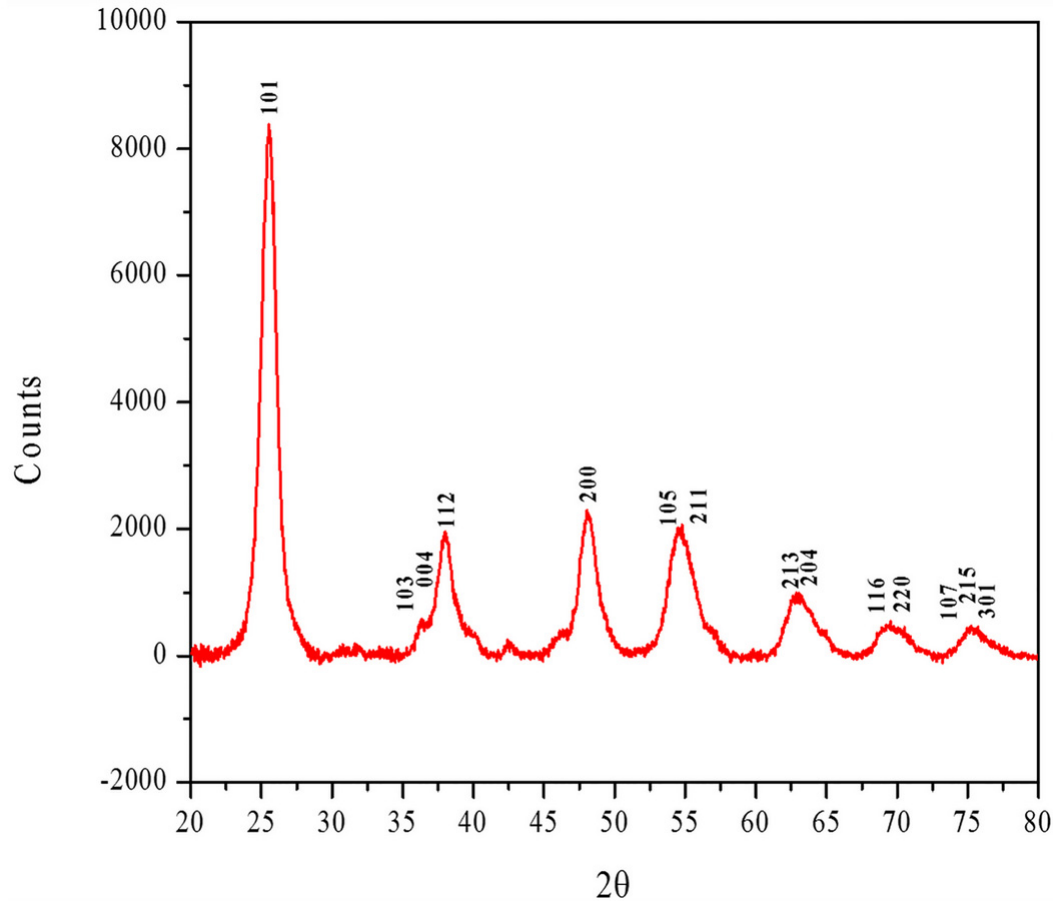
XRD pattern of synthesized TiO_2_ NPs in anatase form.

An x-ray diffraction (XRD) instrument (Siemens D5000) with Cu Kα radiation was employed to analyze the synthesized powder. The XRD pattern of the synthesized nano TiO_2_ is represented in Fig 4. The sharp picks are corresponded to the diffraction pattern of anatase with reference number: 01-073-1764, which confirms the phase of the prepared material.

### Device fabrication

A SiO_2_ wafer is selected as substrate and SU8 polymer waveguide is fabricated on top of that. The waveguide consists of two layers being undercladding and core layers. SU-8 2010 polymer is used for both layers with different concentration of 100% and 70% respectively. The underclad with a thickness of around 12 μm is formed by spin coating and then it is cured at 95°C by soft baking for 2 minutes and post baking for 40 seconds respectively.

**Fig 5:**
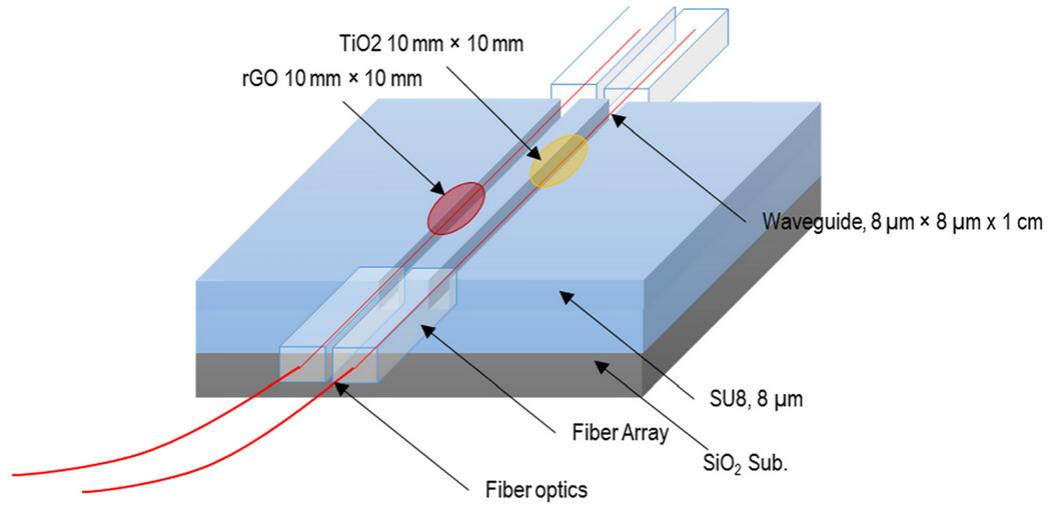
Device structure and dimensions of layer and coatings.

To form the core layer, another SU-8 layer with refractive index of 1.569 (at 1550 nm wavelength) and thickness of 8 μm is spin-coated. The undercladding is patterned by using UV exposure and contact photolithography. Once more, a curing process is carried out at 95°C for 30 minutes. Then the rGO and TiO_2_ dispersed in DI water are drop-casted on two waveguides as illustrated in Fig 5 and treated with UV for 1 hour. Later, fiber arrays are aligned to the channels and the device is ready to use. The waveguide structure, thickness of the layers and dimension of the coatings are stated in this figure as well. Based on our thickness analysis of our 25 samples using drop-casting techniques, we calculated the accuracy of the method to be lower than 50 nm while using other techniques such as plasma deposition, accuracy of ±10 is achievable. However, those methods need expensive machines making the fabrication process challenging.

### Humidity sensing measurement

**Fig 6:**
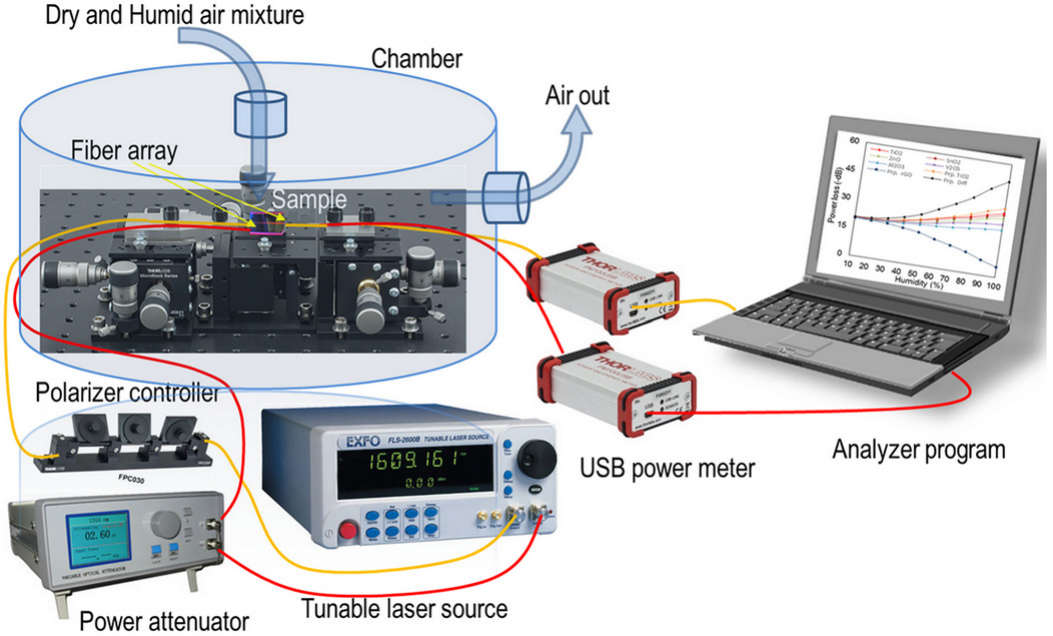
Experimental configuration to control relative humidity inside the chamber and measure the power loss variation at different RHs.

Fig 6 illustrates the experimental configuration of the humidity sensing. A gas tube which provides controlled humidity is placed above the sensing region. Humid nitrogen gas is made by bubbling the dry nitrogen through water. The fellow is controlled by flow meters. The response and recovery times of the proposed device to humid air is measured using a photodiode and a mechanical shutter which is located between the gas source and the sensor. A commercial HI 5862 hydrometer provided by Hanna instruments is used to measure the relative humidity. A polarizer controller (PC) is employed to adjust polarization and to obtain highest extinction ratio. In addition, an optical attenuator is used for power matching between rGO and TiO_2_ coatings. At the output, two USB power meter are used in order to measure the loss of rGO and TiO_2_ channels at the same time during the experiments in different humid conditions. Then, the outputs are processed with analyzer program installed on computer and the charts are plotted.

## Results and Discussion

Mechanism if sensing is straightforward. The electromagnetic field propagation inside the core is not fully confined inside that. A part of the field termed evanescent field can escape the core and propagate inside the cladding. These two fields are related by condition of electromagnetic field continuity on the border of the coating and core the layers. Accordingly, the field power loss inside the core will be affected by any change in the evanescent field [22]. Therefore, if the RH changes, the RI of the cladding will change and thus the power loss in the guided waveguide is affected.

As a result, it is concluded that this field’s strength depends on the refractive index of the core and coating, input field strength and the coating thickness. As a design parameter, therefore, the thickness plays an vital role in sensitivity and performance of the sensor [23]. Thus, different concentrations of the coating have been prepared and used on the sensor and the corresponding performance (response and recovery times) and sensitivity are stated in Tables 1 and 2 for individual and differential sensors respectively. The response and recovery time are calculated based on the maximum recorded values of response and recovery time for both coatings.

**Table 1:** The effect of coating thickness on the sensitivity and response time of the rGO and TiO_2_ sensors.

Sample	Thickness (μm)		Sensitivity (dB/RH%)		Response Time (s)		Recovery time (s)	
	TiO_2_	GO	TiO_2_	GO	TiO_2_	GO	TiO_2_	GO
Sample 1	0.2	0.5	0.09	0.11	0.23	0.17	0.33	0.23
Sample 2	0.7	1.1	0.23	0.23	0.74	0.63	0.91	0.76
Sample 3	0.9	2.3	0.39	0.33	1.83	1.71	2.07	1.93
Sample 4	1.1	3.9	0.505	0.43	2.44	2.33	2.81	2.64

**Table 2:** The effect of the coating thickness on the response time and sensitivity of the differential device.

Sample	Response time (s)	Recovery time (s)	Sensitivity (dB/RH%)
Sample 1	0.23	0.33	0.20
Sample 2	0.74	0.91	0.46
Sample 3	1.83	2.07	0.72
Sample 4	2.44	2.81	0.93

It can be seen that the sensitivity increases with the concentration while the performance decreases. When the coating thickness is too low (Sample 1), it is much less than that of the penetration depth of the evanescent wave. Correspondingly, a lesser number of particles are accessible to interact with the evanescent wave. By increasing the humidity, water molecules that are diffused into the coating layer could interact with a limited number of particles only resulting in a limited absorption of the optical power carried by the evanescent field in the sensing region. Thus, low sensitivity and response time are observed.

On the other hand, in a thick cladding layer, numerous water molecules are diffused and the variation in the refractive index is much which results in high sensitivity. However, the association and dissociation processes of water molecules are timely which causes the recovery and response time to rise. Moreover, the hysteresis of the system increases because of the non-dissociated molecules which are trapped inside the coating layer.

**Fig 7:**
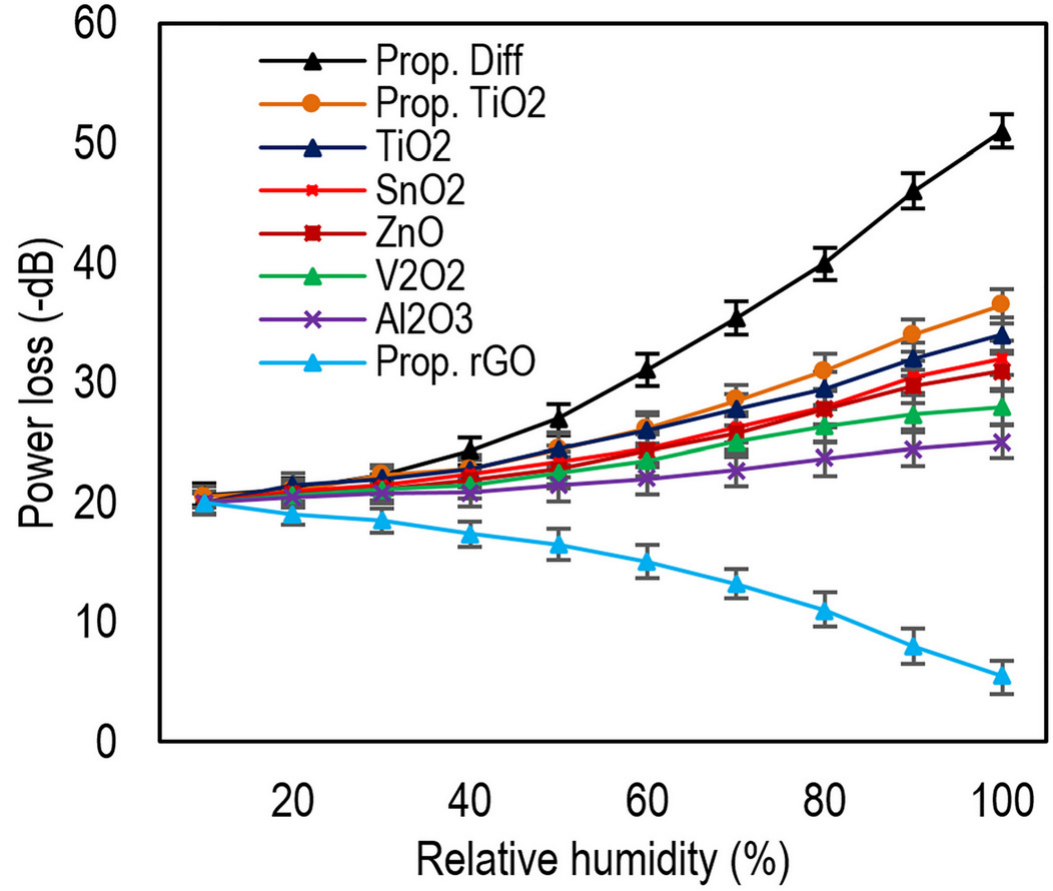
The proposed sensor power loss profile compared to other methods using various coating materials. For detail about other materials preparation please refer [11].

According to Table 2, it is seen that response time of 220 ms is achievable with acceptable sensitivity (Sample 1). However, since high sensitivity and performance are in focus of this study, we further examine the 2^th^ sensor (Sample 2) in the rest of this paper. The power loss profile in respect with RH is illustrated in Fig 7. Moreover, the proposed sensor is compared with other samples with different coating materials. As can be seen, high linearity is observed for wide range of RH between 35 to 98%. Additionally, power loss variation is also very large from 20 to 51 dB for RH in range of 30% to 98%.

In general, if the RH increases, the coating RI increases as well due to water absorption in the coating [11]. This phenomenon substantially changes the boundary condition at guide-coating interface reducing the beam confinement in the core (guide). Therefore, the power loss at the output decreases.

Although this is valid for TiO_2_, the scenario is different for rGO. As the RH increases, the rGO’s refractive index and power loss decreases as can be seen in Fig 7. Water molecules penetrate into the layered structure of the rGO and clusters are formed between their h-bondings and rGO’s epoxy and hydroxyl groups. This process, which is called water swelling, increases the interlayer distance of GO layers. According to reported equation for graphene refracted index *n*_*g*_ [24, 25],

$$n_{g}={\left(\frac{1}{2\omega t_{g}\varepsilon_{0}}\right)}^{1/2}\left[-\sigma_{i}+\sqrt{4\sigma_{r}^{2}+\sigma_{i}^{2}}\right]$$ (1)

with *ω*, *t*_*g*_, *ε*_0_, *σ*_*i*_, *σ*_*r*_ being radian frequency, interlayer distance of graphene layers, permittivity of graphene, and imaginary and real part of graphene’s conductivity respectively, as the interlayer distance increases, the RI decreases and then the loss decreases too.

In contrast, when the rGO sheet is exposed to dry air, water desorption process decreases the interlayer distance (i.e. of rGO films shrinking), and therefore the RI and the conductivity increases and decreases respectively.

At low range of RH between 10 to 35%, water molecules can be absorbed only at the surface of the coating, which causes only slight absorption of light at the coating-air border. Thus, low sensitivity is observed. However, for higher RH range between 35 to 90%, water molecules can penetrate and be adsorbed into the holes and cracks of the coating and form monolayers of water there, which can cause the real part of the coating RI to change. This results in substantial radiation and influences the coating’s transverse guiding. Although primarily, the core’s real part of RI is dominant in the first region, with the increase in RH, the imaginary part may also contribute. Stewart et al. in [26] have reported comparable mechanism in D-fiber-based gas sensor. In our case, the gas can be replaced by water molecules and therefore, the same phenomena could be expected. In the third region above 90%, multilayers of water molecules are formed which results in absorption saturation and sensitivity decrease in comparison with the second region.

**Fig 8:**
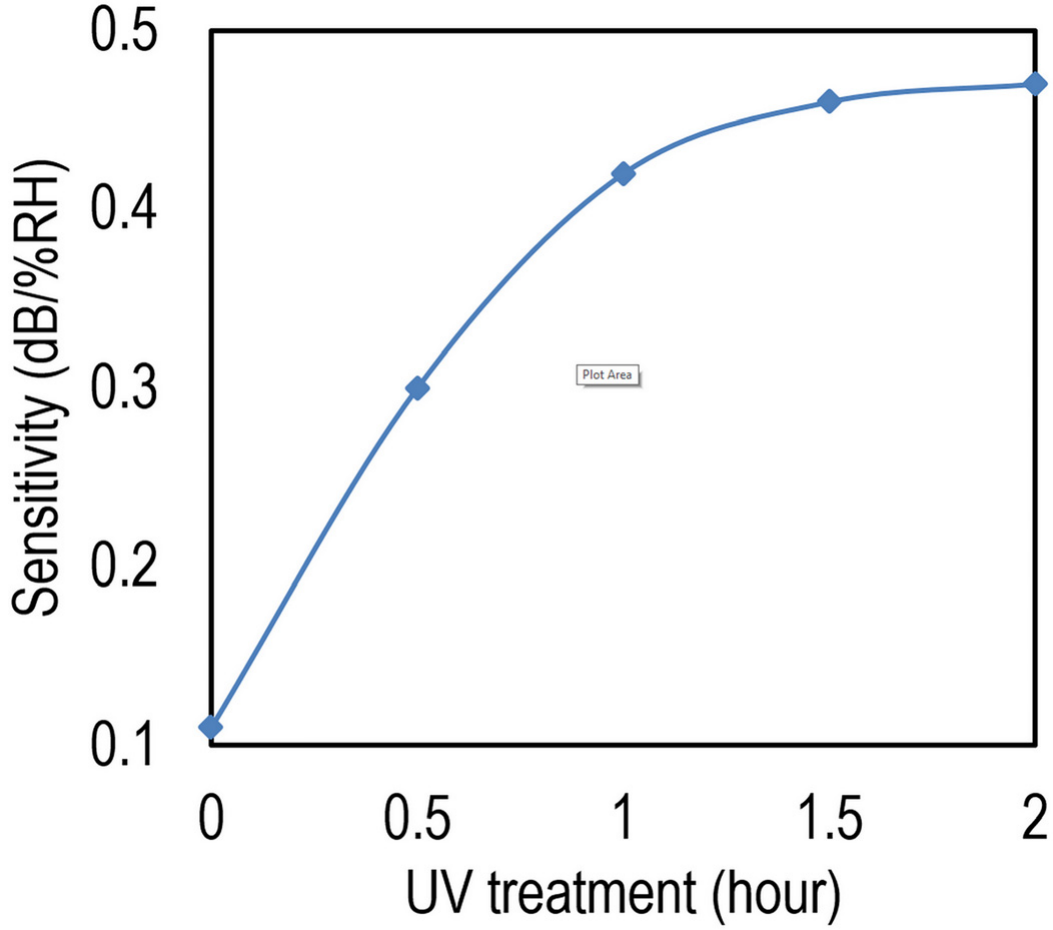
Results of applying UV in order to increase the sensitivity of the proposed device.

The influence of UV exposure duration on sensitivity of TiO_2_ sensor is studied and the results are shown in Fig 8. Apparently, the sensitivity increases as the UV exposure time increases. However, there is a saturation point around 0.47 dB/% RH for exposure more than 90 min.

**Fig 9:**
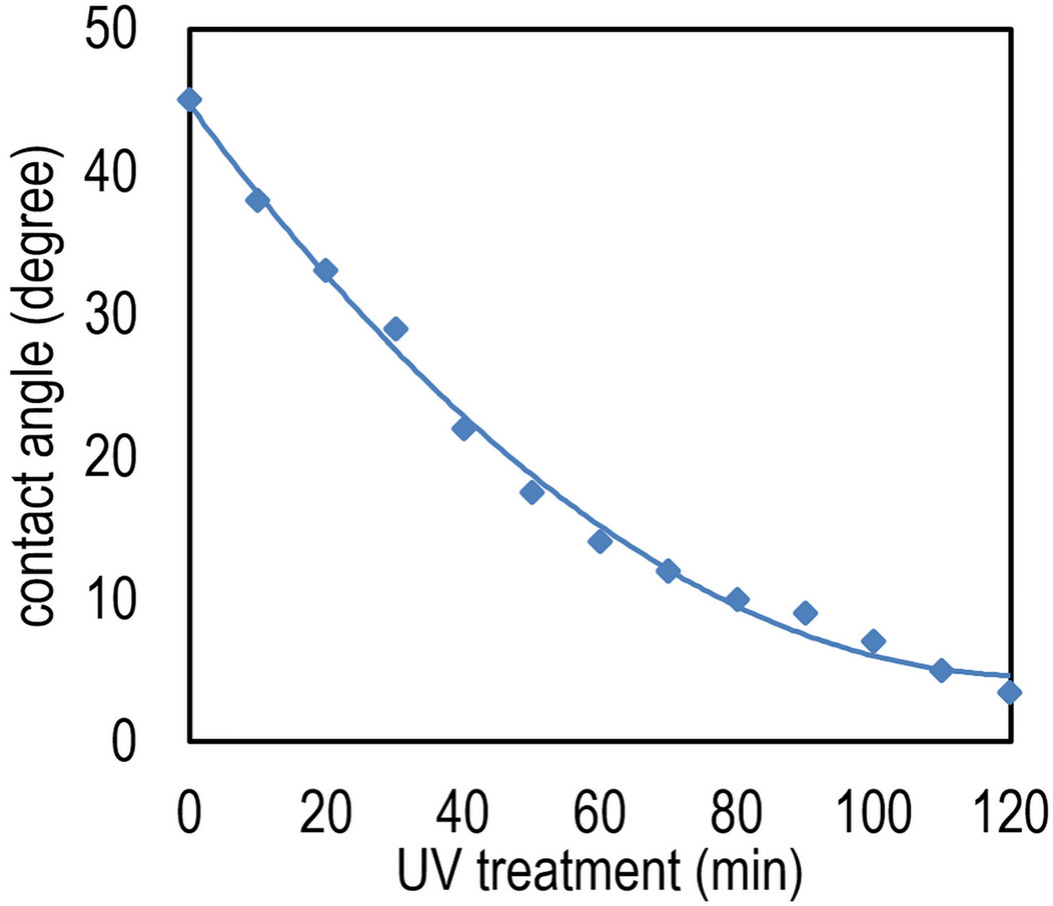
Contact angle in TiO_2_ against UV exposure time.

The effect of UV treatment can be explained by contact angle (CA) concept. The water molecule CA, which is the angle between the tangent line of the liquid phase at the interface of the solid surface and the solid-liquid-gas phases [27] is commonly used to estimate the surface wettability. As shown in Fig 9, the TiO_2_ CA is originally around few tenth of degrees. Due to exposing UV to the surface, the CA can be reduced to almost 0° which means that the surface is highly hydrophilic. The CA has been measured employing a CA-X, Kyowakaimenkagaku CA meter and the UV has been illuminated by a fluorescent black light bulb and an Hg-Xe lamp with intensities of 0.59 and 40 mW/cm^2^ respectively.

**Fig 10:**
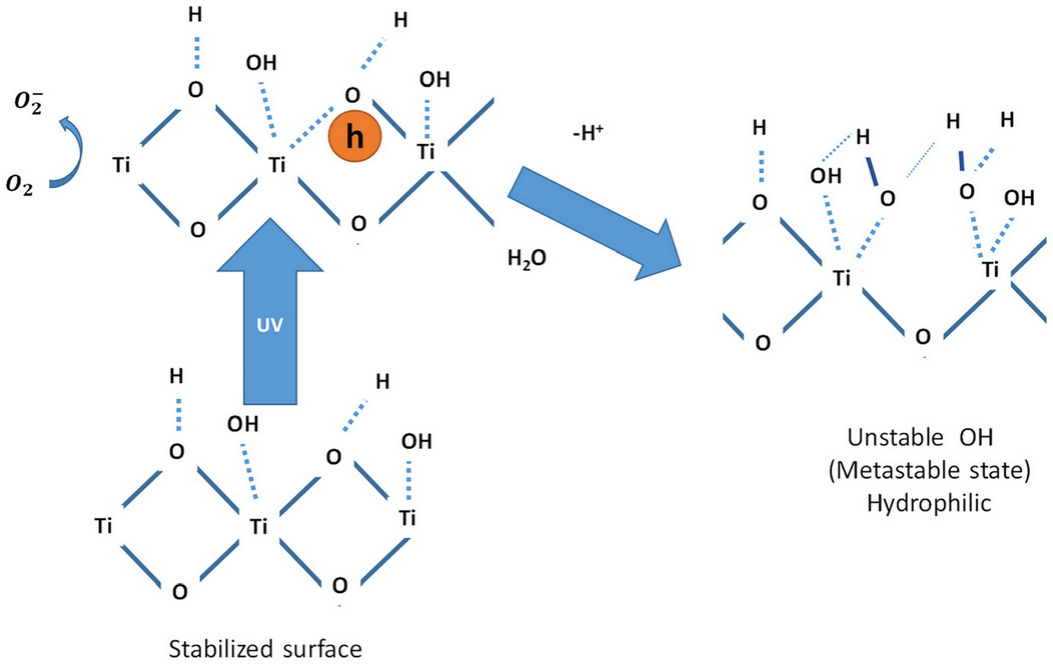
Mechanism of enhancing TiO_2_ hydrophilic properties by exposing UV.

These findings indicate that the hydrophilic properties of the surface is a function of compressive stress which is caused by the surface volume expansion. When TiO_2_ is exposed to UV light, the electron-hole pairs (ehp) are produced and the these photo-generated holes are trapped in the lattice oxygen sites according to [28, 29]. Most of these trapped holes react with the water molecules, which produce OH radicals as illustrated in Fig 10. Nonetheless, a portion of these trapped holes react with TiO_2_ molecules at the titanium site and the bonds between the oxygen ions and the lattice titanium are broken. For the charge compensation, new OH groups are released and the hydrophilic property enhances. Improving the hydrophilic effect in rGO has been studied well in the literature [30] and will not be repeated here again.

**Fig 11:**
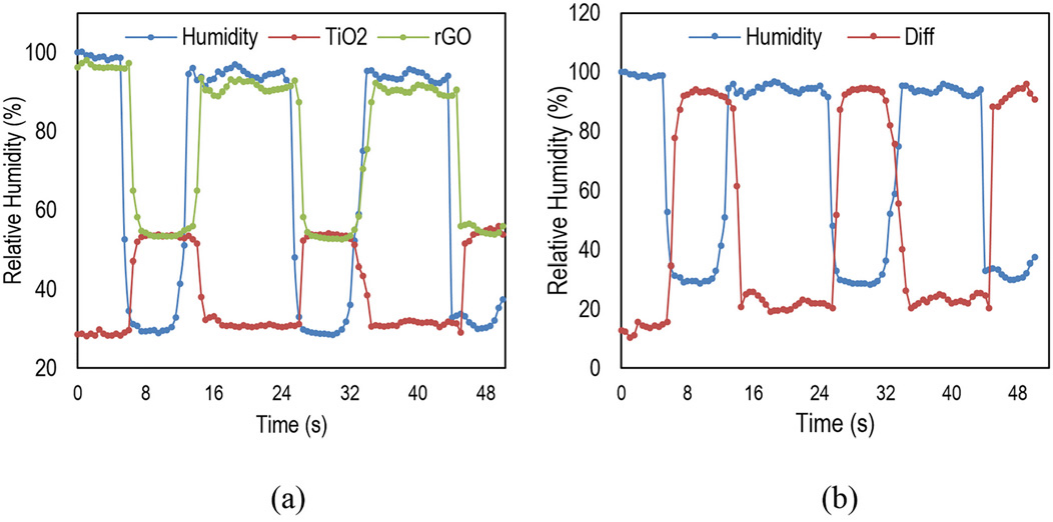
Measured recovery and response and behavior of the proposed humidity sensor rGO and TiO_2_ devices (a); differential device (b).

In order to measure the performance, the device is examined by disrupted humid air supply with predefined time intervals. The results are shown in Fig 11. In subfigure (a) the output is illustrated for TiO_2_ and rGO sensors and in subfigure (b), the results of using the differential device are depicted. As can be see, response and recovery times of around 0.75 and 0.93 sec has been observed respectively. The rapid response of the device is attributed to the quick diffusion of the water molecules and the slower recovery time is due to the slow desorption process.

**Fig 12:**
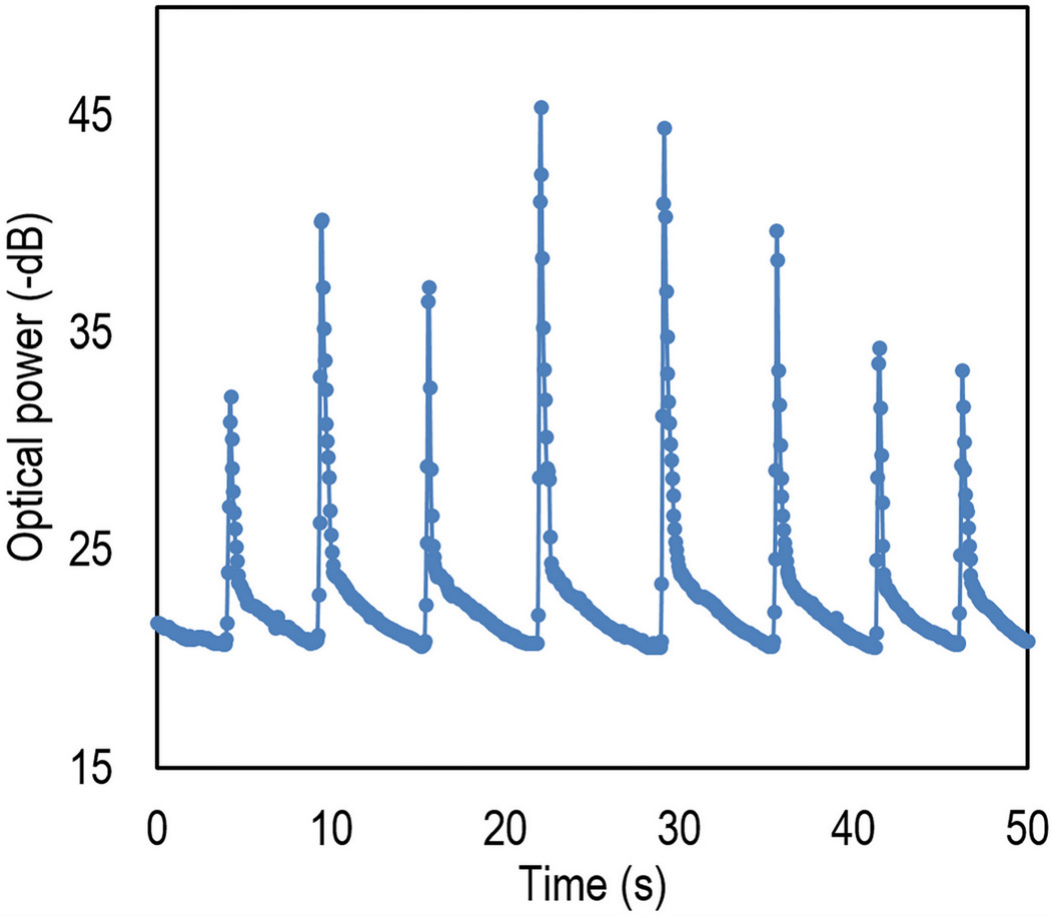
Response of the device to the breathing when the sample is located at a distance of around 10 cm to the human mouth.

To examine the device response time in more detail, specific data are taken and the results are illustrated in Fig 12. Here, breath pulses were applied. As a visional analysis shows, a great reproducibility is resulted. It is worth to mention that the RH is not accurately controlled in this experiment since real breathing is applied however, the range of the humidity is between 50 to 100% RH.

**Fig 13:**
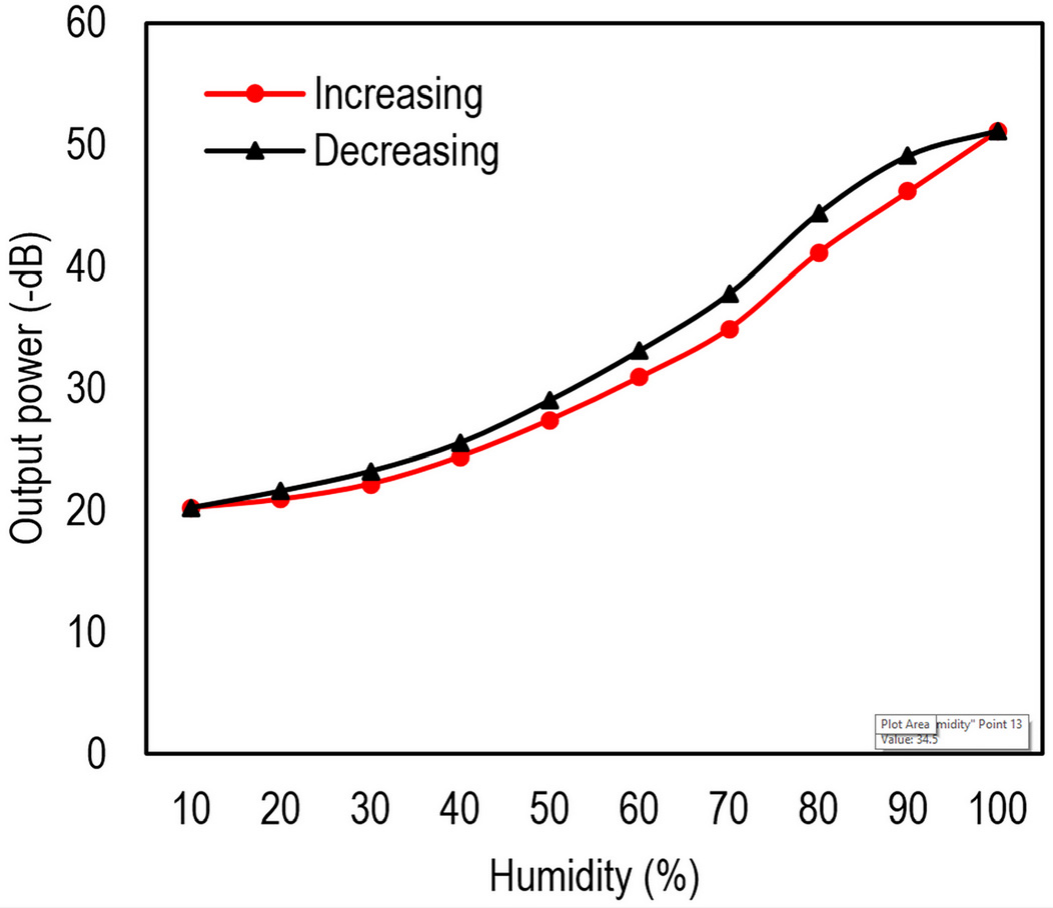
Hysteresis of the proposed sensor for several rising and falling RH cycles in range of 10 to 98% using humidity chamber.

From Fig 12, high repeatability can be reported. Nonetheless, to investigate this parameter perfectly, hysteresis of the device is plotted in Fig 13. According to the results, the hysteresis is roughly ~6% for the worst scenario, which is acceptable in most applications.

**Fig 14:**
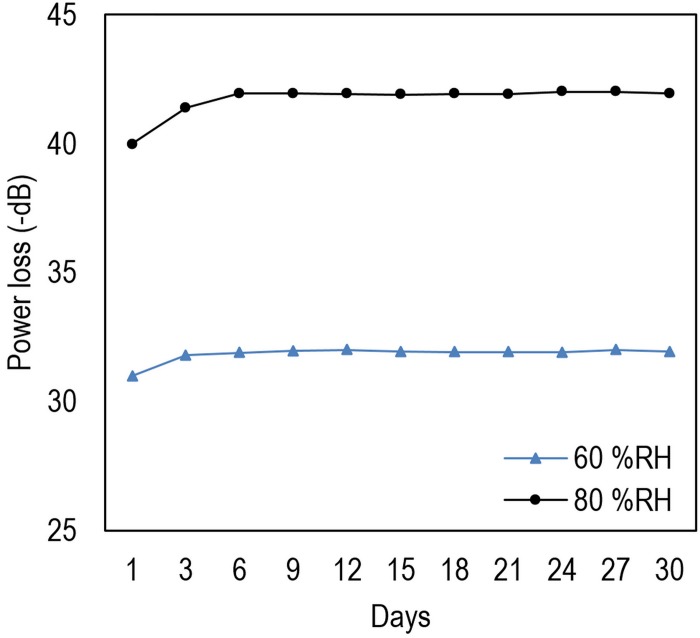
Power loss of the differential sensor for 30 days in 60 and 80%RH.

In addition, to show the repeatability in practice, the device was tested for 30 days at two different RHs and the results are reported in Fig 14. According to the results the power loss increases by 5% for few days and saturates after 3 days. This phenomenon is attributed to dominant water molecules trapped inside the structure of the rGO and TiO_2_. However, the change in power loss is not significant after 3 days.

Furthermore, we investigated the sensor response to several gases such as CO, CO_2_, butane, nitrogen and oxygen, and no significant power variation was observed. As reported in the literature, a low thickness of coating, in range of a nanometer, is normally used for gas sensing [28, 29], while the thickness we used is in the range of micrometers. Furthermore, the coating here is not functionalized and optimized [23] for gas sensing, and not much interaction can therefore be seen between TiO_2_ and gas molecules.

In order to give a comprehensive comparison, the proposed method is compared with other state of the art sensors using different techniques and substrates, which include single mode fiber, (SMF), plastic clad silica fiber (PCS), and gratings such as fiber brag grating (FBG) and long period fiber grating (LPG). These methods employ different coating materials which include Polyvinyl alcohol and TiO_2_ films, Polyimide Gelatin, Hydrogel, TiO_2_, SiO_2_ and ZnO Nanoparticles, Graphene Oxide, and CO polyaniline. Comparison results is reported in Table 3. It shows that the proposed device with 0.47 dB/RH sensitivity in wide range of RH and quick response time is superior compared to others. It is worth to mention that there are sensors with slightly higher sensitivity that this work but the range or/and their performance are not acceptable. In addition, our work uses coating material in solution from making the fabrication process simple and quick while most of the other compared devices use difficult processes.

**Table 3:** Comparison of the proposed sensor with other proposed sensors using different coatings and methods including nanoscale materials.

Reference	Coating material	Substrate	Range	Sensitivity	Resp. time
Gaston et al. [31]	Polyvinyl alcohol film	SMF	70–90%	0.51 dB∕%RH	40 sec
Herrero et al. [32]	TiO2 film	SMF	0–15%	0.49 dB∕%RH	-
Tan et al. [33]	Gelatin	LPG	90–99%	1.2 dB∕%RH	-
Liu et al. [34]	Hydrogel	LPG	38–100%	0.2 dB∕%RH	-
Yeo et al. [35]	Polyimide	FBG	23–97%	5.6 dB∕%RH	>5 min
Aneesh et al. [36]	TiO2 Nano particle	PCS fiber	24–95%	27 mV∕%RH	> 1 sec
Aneesh et al. [37]	ZnO Nanoparticle	PCS	4–96%	0:0012RH−1	> 1 sec
Corres et al. [12]	SiO2 Nanoparticles	SMF	40–98%	0.12 dB%RH	0.15 sec
Lim et al. [38]	Graphene Oxide	SU8 WG	60–100%	0.53 dB%RH	~1 sec
Vijayan et al. [6]	Co polyaniline	Optical fiber	20%-95%	6 mV/%RH	~1 min
Proposed rGO	GO solution	SU8 WG	35–95%	0.22 dB/%RH	0.68 sec
Proposed TiO2	Nano anatas TiO2	SU8 WG	30–95%	0.25 dB/%RH	0.75 sec
Proposed Differential	GO + TiO2	Su8 WG	35–95%	0.47 dB/%RH	0.75 sec

In this design, we used two photo detection devices containing two analog amplifiers and two analog digital converters which would be costly in practice. However, in practical implementation, a CMOS differential amplifier (Diff-Amp) could be designed to amplify the difference between two outputs. In this way, the system can be designed using one amplifier and one analog digital converter to reduce the cost and complexity.

## Conclusion

An optical humidity sensor was presented based on the effect of evanescent field in anastase TiO_2_ nanoparticle and reduced graphene oxide coatings. The approach to prepare the active material, results in in TiO_2_ in solution form making the fabrication process fast, easy and cheap. The sensor sensitivity and performance were extensively investigated at different humid conditions. It was observed that the device response is almost linear over a wide dynamic range (35%–98% RH) with high sensitivity of around 0.21 dB/%RH. In addition, the effect of several parameters such as coating thickness and the UV treatment time were studied on repeatability, performance and sensitivity of the device and the attributed mechanisms explained. It was revealed that by increasing the thickness, the sensitivity increases while the performance decreases. With high sensitivity and low response time of ~0.7 s for humidification and 0.95 sec for desiccation, the device is most suitable for real time human breath monitoring application. It is believed that the approach could be used for other applications such as PH meter, temperature sensor and glucose concentration sensing.
